# Commercial determinants of health revisited: integrating business scholarship for greater public health impact

**DOI:** 10.1186/s12992-026-01187-y

**Published:** 2026-01-30

**Authors:** Junghoon Park, Bryan W. Husted

**Affiliations:** 1 College of Business Administration, Loyola Marymount University, 1 LMU Drive, Los Angeles, CA 90045 USA; 2 EGADE Business School, Tecnológico de Monterrey, Av. Rufino Tamayo y Av. Eugenio Garza Lagüera, Valle Oriente, San Pedro Garza García, Nuevo León 66269 México

**Keywords:** Commercial determinants of health, CDOH, Interdisciplinary research, Business scholarship, Management studies

## Abstract

In her influential 2020 review in *Globalization and Health*, Mialon synthesizes the commercial determinants of health (CDOH) literature and underscores how commercial actors are connected to public health through the institutional environments in which they operate. Much of this research conceptualizes firms as institutionally embedded but relatively homogeneous actors, emphasizing external practices such as the production and promotion of harmful commodities, lobbying, and corporate social responsibility initiatives. While recent CDOH scholarship has begun to recognize the importance of organizational-level factors, few studies analyze the internal processes through which corporate conduct with public health consequences emerges. We argue that integrating insights from management scholarship can enrich CDOH research by opening the “black box” of the firm and clarifying how such conduct arises from interactions among institutional contexts, organizational arrangements, and individual-level dynamics. Drawing on management research, we conceptualize firms as multilevel systems in which institutional conditions inform conduct through organizational governance, resources, and practices, as well as through individual values, cognitions, sensemaking, and interaction among organizational members and stakeholders. These analytically distinct yet dynamically interconnected levels give rise to patterned forms of corporate conduct that affect public health. By foregrounding these multilevel connections, this paper advances a management-informed framework for CDOH research that moves beyond documenting harmful practices toward explaining how firms contribute to public health outcomes and identifying leverage points for intervention.

## Clinical Trial Number

Not applicable. This manuscript is a conceptual paper and does not involve clinical trials.

## Introduction

Commercial determinants of health (CDOH) research illuminates the systems, practices, and pathways through which commercial actors influence health and equity [1]. In her widely cited 2020 review in *Globalization and Health* [2], Mialon synthesizes this literature by identifying three interconnected domains: the production and promotion of unhealthy commodities; business, market, and political practices that are harmful to health; and global drivers of ill-health, including trade liberalization and globalization. Drawing on the concept of the “industrial epidemic,” her review underscores how corporate activity contributes to persistent health harms, linking business systems to public health outcomes [1–7].

Much of the CDOH literature has conceptualized firms as institutionally embedded but relatively homogeneous actors [5]. It examines corporate engagement with public health primarily through practices such as lobbying, marketing, the strategic use of corporate social responsibility initiatives, and the management of supply chains associated with the production and distribution of harmful commodities [1]. Although this literature has advanced understanding of how commercial actors are associated with public health outcomes via systemic political and economic mechanisms, it has paid less attention to the internal dynamics underlying corporate behavior. Recent CDOH research has begun to move beyond this view by recognizing that corporate influence also depends on organizational-level factors such as governance systems, practices, resources, and transparency [4–6]. However, the ways firms interpret, operationalize, and embed these organizational arrangements into internal norms, routines, and decision processes—and how these processes interact across levels—have received limited attention. Without examining these dynamics, CDOH research risks obscuring why similarly positioned firms respond differently to comparable institutional environments and why health-harming commercial practices persist over time. This limits the literature’s ability to move beyond descriptive accounts toward explaining the emergence of corporate behaviors and, in turn, identify at which levels interventions are most likely to yield public health improvements.

In this paper, we argue that integrating insights from business scholarship, particularly management research, can help address this gap by opening the “black box” of the firm—shedding light on firms as multilevel systems. Management research examines how firms are structured and the ways decisions unfold under real-world constraints [8–12]. It also reveals organizational heterogeneity, helping explain why firms facing similar institutional pressures adopt divergent strategies with distinct health consequences and how micro-level behaviors, such as managerial incentives and employee practices, connect to population-level outcomes.

We conceptualize firms as multilevel systems encompassing: (a) institutional-level factors that capture the economic, political, and social environments within which firms operate; (b) organizational-level factors that reflect firms’ internal governance systems, resources, and practices; and (c) individual-level factors such as the values, cognitions, and sensemaking of organizational members and their interactions with stakeholders. Viewing firms through this lens clarifies how corporate activity and public health are connected via interacting processes across levels and directs attention to internal mechanisms—such as governance, culture, accountability, and innovation—that can sustain or disrupt harmful practices.

Building on broader developments in public health, this integration parallels the social determinants of health literature, in which scholars increasingly examine how institutional arrangements and policy decisions distribute power and configure structural environments [13, 14]. As this scholarship shifts toward institutional and governance systems, firms emerge as multilevel systems whose internal dynamics and behavioral processes warrant systematic examination. Management scholarship strengthens this examination by illuminating how organizational learning, change processes, and leadership structures shape the persistence or transformation of health-harming practices, and by clarifying where levers at each level are most likely to improve public health outcomes.

## Opening the black box of the firm: a multilevel perspective

CDOH research has shed light on the structural, political, and economic systems through which firms influence public health—often via outward-facing commercial practices—yet firms themselves remain a “black box”: the literature examines what firms do but less so the internal processes through which that conduct arises. Management research offers a complementary perspective by conceptualizing firms as multilevel systems in which institutional-, organizational-, and individual-level factors interact in interdependent ways [8–12, 15]. This conceptual lens clarifies how interactions across levels give rise to patterns of corporate conduct affecting health outcomes.

At the institutional level, management scholarship investigates how broader economic, political, and social environments are associated with corporate behavior [16]. These environments create incentives and constraints that influence corporate priorities and strategies [17]. CDOH research likewise examines how global economic, political, and regulatory systems create conditions under which harmful commercial practices and products persist. However, this work has paid less attention to how firms internally interpret and respond to such institutional conditions. Management scholarship provides insights into why and how firms vary in their responses—from passive conformity to active resistance or manipulation—depending on the nature of institutional conditions and firm-level interests and constraints [18].

At the organizational level, management scholarship examines how firms interpret and implement institutional conditions through internal governance systems, resources, and practices [19]. These organizational-level factors are central to how firms coordinate activities, allocate resources, and balance competing objectives under broader institutional conditions. Management research views these systems not only as administrative mechanisms but also as interpretive frameworks through which firms make sense of and respond to institutional conditions [20]—including, in the context of CDOH, expectations concerning public health. Viewed in this way, firms are understood as dynamic systems that mediate the integration of institutional contexts and individual-level dynamics [15], shaping corporate conduct with public health consequences.

At the individual level, management scholarship studies how the values, cognitions, and sensemaking of organizational members—and their interactions with stakeholders—inform how organizational practices are developed and implemented [21]. Individual-level mechanisms—such as motives, identities, and moral reasoning among organizational members at different hierarchical positions—condition how firms interpret and act upon institutional expectations [22]. Often described as microfoundations, management scholarship emphasizes that patterns of corporate conduct emerge from the actions and interactions of individuals whose judgments are embedded in social and relational contexts [23, 24]. Group interactions, shared identities, and interpersonal relationships provide the settings through which meaning is constructed and coordinated action becomes possible within organizations [25]. Integrating this level into CDOH inquiry underscores that corporate conduct influencing health outcomes emerges not solely from institutional conditions or organizational arrangements, but also from how individuals interpret, evaluate, and act on those conditions in practice.

To clarify the analytical contributions of each level to CDOH research, Table 1 summarizes the key features of this framework.

**Table 1:** A multilevel perspective for integrating management scholarship into CDOH research

Level	Focus of Analysis	Representative Management Subfields	Relevance for CDOH and Public Health
Institutional	Broader economic, political, and social environments that create the incentives and constraints shaping corporate priorities and strategies	Organizational Theory; Strategic Management; International Business; Business and Society; Business Ethics	Illuminates how regulatory systems, market governance structures, and cultural norms distribute authority and accountability, establishing the institutional conditions under which firms interpret and respond to public health expectations.
Organizational	Internal governance systems, resource allocation, and practices through which firms interpret and implement institutional conditions in corporate conduct	Organizational Theory; Strategic Management; International Business; Business and Society; Business Ethics	Explains how organizational structures and coordination mechanisms mediate institutional conditions, influencing the ways that corporate practices produce varied public health outcomes.
Individual	Values, cognitions, sensemaking, and interpersonal interactions among organizational members and stakeholders	Organizational Behavior; Business Ethics	Reveals how individual interpretation, moral reasoning, and interactions with stakeholders inform corporate conduct, thereby contributing to patterns that affect public health outcomes.

## Cross-level connections: illustrations from management research

The institutional, organizational, and individual dimensions together constitute an integrated multilevel system through which corporate conduct with public health implications takes shape. In this section, we draw on exemplary management studies at the intersection of business and public health that demonstrate cross-level connections. We first examine studies that address institutional-organizational connections and then turn to research that incorporates all three levels.

### Institutional-organizational linkages

Recent management scholarship reinforces the importance of examining cross-level dynamics to understand how corporate practices influencing health emerge and evolve. Rietveld and Patel [26] provide a vivid illustration in their study of antiretroviral therapy coverage among low- and middle-income countries, identifying a reinforcing relationship whereby improved health access fosters entrepreneurship and economic development, which in turn strengthens health infrastructure. This dynamic illustrates how institutional environments and organizational activities co-evolve, demonstrating bidirectional influence between business activities and public health. Another example is offered by Park and colleagues [27], who conceptualize improving public health as a grand challenge that demands coordinated, multilevel action across institutional and organizational boundaries. Their framework shows how organizational constraints—such as resource tensions, information asymmetries, and short-term profit pressures—and institutional contexts—including regulatory unevenness, governance capacity, and sociocultural barriers—condition firms’ ability to engage with public health challenges and the consequences that follow. Together, these studies show that corporate conduct affecting public health outcomes reflects not only internal organizational systems but also the interaction of those systems with institutional conditions.

A related perspective on these dynamics is offered by Montiel and colleagues [28], who examine how multinationals’ cross-border activities influence the emergence and spread of communicable diseases. Their analysis links disease vulnerability to international business contextual factors—such as host-country regulatory quality, urbanization, trade liberalization, and global migration—as well as to multinationals’ activities, including foreign direct investment, corporate political activity, global supply chain management, and international travel. For example, multinationals’ labor-seeking investment in host countries may be associated with overcrowded and unsafe working conditions that heighten disease transmission risks, while resource-seeking investment—such as mining and large-scale agriculture—can degrade ecosystems and increase opportunities for zoonotic spillover. Corporate political activity and supply chain integration can further facilitate the transnational spread of infectious diseases. In sum, the study demonstrates how institutional conditions and organizational factors jointly produce public health risks that offshore morbidity and scale globally, underscoring the importance of analyzing the interaction between institutional and organizational dynamics.

Research on institutional and organizational mechanisms of responsibility indicates that the allocation of authority and responsibility within firms and across supply chains critically affects firms’ capacity to prevent health harms. Caulfield and Lynn [29] conceptualize corporate responsibility as a federated system in which authority for social and ethical commitments is distributed and constitutionally constrained across multiple organizational units rather than centralized in a single locus of control. Their analysis of the Rana Plaza factory collapse and the subsequent Bangladesh Accord finds that, despite firms’ public commitments to labor and safety standards, weakly coordinated and informally governed responsibility arrangements across headquarters, sourcing teams, suppliers, and auditing intermediaries undermined efforts at preventing health harms. In contrast, the Accord suggests how formalized power-sharing arrangements—anchored in binding agreements among firms, unions, and civil society—can enhance accountability and workplace safety. Overall, the study underscores that broader public health effects depend not only on firms’ stated commitments, but also on the ways that authority and responsibility are organized and enforced within and across organizational boundaries.

### Institutional-organizational-individual linkages

Beyond this two-level nexus, a growing body of management research examines the interaction of individual-level mechanisms with institutional and organizational conditions, clarifying how firms develop and execute practices with public health relevance. Girschik [30] examines this connection through an analysis of how corporate actors manage legitimacy in business-driven social change via relational work, drawing on Novo Nordisk’s efforts to improve diabetes care in Indonesia. Her analysis finds that legitimacy building is not merely a structural or organizational process; it is accomplished through boundary-spanning individuals who interpret societal expectations and translate them into organizational action. By framing, negotiating, and redefining roles in dialogue with government officials, medical professionals, and other nonmarket actors, these individuals connect institutional expectations with organizational practices. This study shows how micro-level interaction and sensemaking link individual interpretation to organizational legitimacy processes and broader institutional arrangements.

In a similar vein, Van Cranenburgh and Arenas [31] study how moral reasoning guides individual decision-making in health-related corporate initiatives. Their study of the Heineken Africa Foundation uncovers the strategic and ethical dilemmas faced by managers implementing philanthropic and health programs in Sub-Saharan Africa, where institutional voids provide limited external guidance on firms’ social and health-related roles. The analysis shows that managers’ moral reasoning—grounded in principles of duty, fairness, and care—can motivate engagement with public health challenges in contexts where formal institutional direction is weak or absent. These individual-level judgments are reflected in organizational choices concerning the design and governance of corporate health programs, contributing to emerging expectations about firms’ roles in addressing public health challenges within institutionally fragile environments.

Lastly, Dumalanède and colleagues [32] analyze Unjani Clinics, a social-franchise network in South Africa, to examine how leadership and governance mechanisms foster equitable health care delivery under challenging institutional conditions. They identify network stewardship—a relational and value-based form of leadership—as a key mechanism that aligns individual motivation and identity with organizational goals. Through mentorship, shared purpose, and informal spaces for care, leaders cultivate trust and autonomy among nurse-franchisees, mitigating mission conflict and sustaining commitment to health equity. These dynamics illustrate how individual motivation and relational leadership practices stabilize organizational systems and support the provision of health services under challenging institutional conditions.

Taken together, these management studies indicate that corporate conduct with public health relevance arises from ongoing interactions across multiple, nested levels of governance and action, rather than from isolated decisions. Understanding these connections helps explain why firms differ in their engagement with public health challenges and where interventions are most likely to be effective.

## Integrating a multilevel perspective into CDOH research

A multilevel perspective has long been central to management scholarship [8–12, 15]. Integrating this perspective into CDOH research offers a systematic way to analyze how corporate conduct with public health consequences emerges from cross-level dynamics, rather than treating firms as homogeneous actors.

Conceptually, management scholarship helps unpack how core concerns in CDOH—such as corporate power, influence, and accountability—are organized and expressed within firms, rather than being treated solely at the level of markets, policies, or institutional systems. It does so by specifying how these concerns emerge through cross-level interactions involving organizational members and stakeholders. Management research shows that power is expressed through governance arrangements that allocate decision-making authority under prevailing institutional rules and expectations; influence operates through firms’ control over critical resources and dependence on key relationships; and accountability is sustained—or undermined—through the legitimacy strategies and interpretive judgments organizational members use to justify corporate actions to stakeholders. Examining these dynamics helps identify levers at each level through which corporate conduct with public health relevance is produced, including ownership structures, board oversight, coordination mechanisms, and managerial sensemaking. It also points to avenues for change, such as redesigning governance systems, strengthening transparency and accountability arrangements, and fostering interpretive capacities that embed social and public health considerations into corporate decision-making.

Methodologically, multilevel and longitudinal qualitative designs, process tracing, comparative case studies, and configurational analyses enable scholars to examine how institutional conditions are interpreted, negotiated, and translated into practice through organizational structures, routines, and individual-level dynamics. Together, these approaches move analysis beyond documenting associations between corporate activities and public health toward examining the situated processes through which interacting structures, practices, and actors affect the production of corporate practices influencing health outcomes.

In brief, management scholarship invites CDOH scholars to examine firms as multilevel systems. Such understanding does not excuse harmful practices; rather, it enables analysis of how corporate conduct can be reoriented to better support public health.

## Concluding remarks

In this paper, we respond to Mialon’s 2020 review of the CDOH [2] and recent developments in this literature [1, 3–7] by proposing a constructive extension that integrates insights from management scholarship. While CDOH research has shed light on how business systems are linked to public health through economic and political mechanisms, it has given limited attention to the internal processes through which firms interpret, operationalize, and act on those influences. Our contribution advances this trajectory by opening the “black box” of the firm—shifting analytical attention from *what* corporations do toward *how* and *why* they do it. Understanding the CDOH therefore requires examining firms as multilevel systems in which institutional-, organizational-, and individual-level factors combine in the production of corporate conduct and its associated public health consequences.

We argue that management scholarship offers conceptual and methodological lenses that enhance the analysis of firms as multilevel systems in ways directly relevant to CDOH and public health research agendas. Integrating management insights does not replace the normative commitments of public health scholarship. It invites CDOH research to move beyond documenting harmful practices toward explaining the organizational and institutional processes that give rise to them and identifying the conditions under which meaningful improvements in public health become possible. In line with recent calls to govern, not merely document, the structures that sustain commercial determinants [7], we suggest that improving public health requires engaging with the governance and management of firms themselves. A management-informed multilevel perspective thus offers a structured foundation for the next phase of CDOH research.

Looking ahead, we encourage deeper interdisciplinary collaboration that links institutional contexts with firms’ internal dynamics, clarifying how corporate activity affects health outcomes and under what conditions it can be reoriented to better support public health. Management scholarship’s multilevel perspective can help advance the CDOH agenda and strengthen its capacity to inform interventions aimed at aligning business practices with public health goals.

## Data Availability

No datasets were generated or analysed during the current study.

## Abbreviations

CDOH: Commercial Determinants of Health

## References

[1] Kickbusch I, Allen L, Franz C. The commercial determinants of health. Lancet Glob Health. 2016;4(12):e895–6. 27855860 10.1016/S2214-109X(16)30217-0

[2] Mialon M. An overview of the commercial determinants of health. Glob Health. 2020;16(1):74. 10.1186/s12992-020-00607-x PMC7433173 32807183

[3] Friel S, Collin J, Daube M, Depoux A, Freudenberg N, Gilmore AB, et al. Commercial determinants of health: future directions. Lancet. 2023;401(10383):1229–40. 36966784 10.1016/S0140-6736(23)00011-9

[4] Gilmore AB, Fabbri A, Baum F, Bertscher A, Bondy K, Chang HJ, et al. Defining and conceptualising the commercial determinants of health. Lancet. 2023;401(10383):1194–213. 36966782 10.1016/S0140-6736(23)00013-2

[5] Lacy-Nichols J, Nandi S, Mialon M, McCambridge J, Lee K, Jones A, et al. Conceptualising commercial entities in public health: beyond unhealthy commodities and transnational corporations. Lancet. 2023;401(10383):1214–28. 36966783 10.1016/S0140-6736(23)00012-0

[6] Burgess R, Nyhan K, Freudenberg N, Ransome Y. Corporate activities that influence population health: a scoping review and qualitative synthesis to develop the HEALTH-CORP typology. Glob Health. 2024;20(1):77. 10.1186/s12992-024-01082-4 PMC11549802 39516852

[7] Maani N, Petticrew M, Galea S. The commercial determinants of health. New York: Oxford Univ; 2023.

[8] Hitt MA, Beamish PW, Jackson SE, Mathieu JE. Building theoretical and empirical bridges across levels: multilevel research in management. Acad Manag J. 2007;50(6):1385–99.

[9] Aguinis H, Glavas A. What we know and don’t know about corporate social responsibility: a review and research agenda. J Manag. 2012;38(4):932–68.

[10] Aguilera RV, Rupp DE, Williams CA, Ganapathi J. Putting the S back in corporate social responsibility: a multilevel theory of social change in organizations. Acad Manag Rev. 2007;32(3):836–63.

[11] George G, Howard-Grenville J, Joshi A, Tihanyi L. Understanding and tackling societal grand challenges through management research. Acad Manag J. 2016;59(6):1880–95.

[12] Stahl GK, Filatotchev I, Ireland RD, Miska C. Five decades of research on the role of context in management: from universalism toward contingent, multilevel and polycontextual perspectives. Acad Manag Collect. 2023;2(1):1–18.

[13] Braveman P, Egerter S, Williams DR. The social determinants of health: coming of age. Annu Rev Public Health. 2011;32:381–98. 21091195 10.1146/annurev-publhealth-031210-101218

[14] Braveman P, Gottlieb L. The social determinants of health: it’s time to consider the causes of the causes. Public Health Rep. 2014;129(Suppl 2):19–31. 24385661 10.1177/00333549141291S206 PMC3863696

[15] Cowen AP, Rink F, Cuypers IRP, Grégoire DA, Weller I. Applying Coleman’s boat in management research: opportunities and challenges in bridging macro and micro theory. Acad Manag J. 2022;65(1):1–10.

[16] Greenwood R, Oliver C, Suddaby R, Sahlin-Andersson K, editors. The SAGE handbook of organizational institutionalism. London: SAGE; 2008.

[17] Oliver C. Sustainable competitive advantage: combining institutional and resource-based views. Strateg Manag J. 1997;18(9):697–713.

[18] Oliver C. Strategic responses to institutional processes. Acad Manag Rev. 1991;16(1):145–79.

[19] Daft RL, Weick KE. Toward a model of organizations as interpretation systems. Acad Manag Rev. 1984;9(2):284–95.

[20] Besharov ML, Smith WK. Multiple institutional logics in organizations: explaining their varied nature and implications. Acad Manag Rev. 2014;39(3):364–81.

[21] Porter LW, Schneider B. What was, what is, and what may be in OP/OB. Annu Rev Organ Psychol Organ Behav. 2014;1:1–21.

[22] Gond JP, El Akremi A, Swaen V, Babu N. The psychological microfoundations of corporate social responsibility: a person-centric systematic review. J Organ Behav. 2017;38(2):225–46.

[23] Felin T, Foss NJ, Ployhart RE. The microfoundations movement in strategy and organization theory. Acad Manag Ann. 2015;9(1):575–632.

[24] Barney J, Felin T. What are microfoundations? Acad Manag Perspect. 2013;27(2):138–55.

[25] Heath C, Sitkin SB. Big-B versus Big-O: what is organizational about organizational behavior? J Organ Behav. 2001;22(1):43–58.

[26] Rietveld CA, Patel PC. Antiretroviral therapy coverage, entrepreneurship, and development in low- and middle-income countries. Bus Soc. 2025;64(3):441–71.

[27] Park J, Montiel I, Husted BW, Balarezo R. The grand challenge of human health: a review and an urgent call for business–health research. Bus Soc. 2022;61(5):1353–415.

[28] Montiel I, Park J, Husted BW, Velez-Calle A. Tracing the connections between international business and communicable diseases. J Int Bus Stud. 2022;53(8):1785–804. 35345569 10.1057/s41267-022-00512-y PMC8942389

[29] Caulfield M, Lynn A. Federated corporate social responsibility: constraining the responsible corporation. Acad Manag Rev. 2024;49(1):32–55.

[30] Girschik V. Managing legitimacy in business-driven social change: the role of relational work. J Manag Stud. 2020;57(4):775–804.

[31] Van Cranenburgh K, Arenas D. Strategic and moral dilemmas of corporate philanthropy in developing countries: heineken in Sub-Saharan Africa. J Bus Ethics. 2014;122(3):523–36.

[32] Dumalanède C, Ciambotti G, Lashitew AA. Addressing health care inequality through social franchising: the role of network stewardship in impact intermediation. Bus Soc. 2025;64(3):521–57.

